# Do alcohol industry-funded organisations act to correct misinformation? A qualitative study of pregnancy and infant health content following independent analysis

**DOI:** 10.1186/s12992-025-01125-4

**Published:** 2025-11-12

**Authors:** Gemma Mitchell, Chris Baker, May CI van Schalkwyk, Nason Maani, Mark Petticrew

**Affiliations:** 1 Institute for Social Marketing and Health, Faculty of Health Sciences and Sport, University of Stirling, Stirling, FK9 4LA UK; 2 Department of Non-Communicable Disease Epidemiology, Faculty of Epidemiology and Public Health, London School of Hygiene and Tropical Medicine, 15-17 Tavistock Place, London, WC1H 9SH UK; 3 Present Address: Centre for Pesticide Suicide Prevention, University of Edinburgh, Edinburgh, WC1H 9SH UK; 4 Global Health Policy Unit, School of Social and Political Science, College of Arts, Humanities and Social Sciences, University of Edinburgh, 15a George Square, Edinburgh, EH8 9LD UK; 5 Department of Public Health, Environments and Society, Faculty of Public Health and Policy, London School of Hygiene and Tropical Medicine, 15-17 Tavistock Place, London, WC1H 9SH UK

**Keywords:** Health promotion, Misinformation, Alcohol, Pregnancy and infant health, Alcohol industry, Commercial determinants of health, Corporate political activity, Corporate social responsibility

## Abstract

**Background:**

Access to reliable, accurate, and up-to-date health information is a crucial component of global population health. Like other health-harming industries, the alcohol industry is known to provide misinformation to the public, including on alcohol, pregnancy, and infant health. It is unknown whether industry information changes following independent public health analysis.

**Methods:**

We extracted data using the homepage, menu, and search tool functions (where available) from seven industry-funded charity and nonprofit company websites (Aware, South Africa; Drinkaware, Ireland; Drinkaware, United Kingdom; Éduc’alcool, Canada; DrinkWise, Australia; Foundation for Advancing Alcohol Responsibility, United States; and International Alliance for Responsible Drinking) that have previously been found to misrepresent the evidence on alcohol, pregnancy, and infant health. We conducted a qualitative, thematic analysis using a published framework of ‘dark nudges and sludge’ misinformation techniques.

**Results:**

Omission of information, functionality problems, and the positioning and sequencing of information in ways that framed or obfuscated its meaning were the most common forms of misinformation identified. These types of misinformation were often mixed with (limited) relevant information and were most often found in combination. We found pregnancy and infant health information for the consumer on five of the seven websites studied (Drinkaware, Ireland; Drinkaware, United Kingdom; DrinkWise; Éduc’alcool; and Aware). Information on pregnancy and fetal alcohol spectrum disorder was found on these five sites, although they did not all provide information on miscarriage, breastfeeding, or fertility. We could not find any pregnancy and infant health information directed to the consumer on the remaining sites (Foundation for Advancing Alcohol Responsibility and International Alliance for Responsible Drinking). Six of the seven websites had a search tool function; these often produced irrelevant information.

**Conclusions:**

Following independent public health analysis of their informational outputs, misinformation about pregnancy and infant health remains present on alcohol industry-funded websites. Warnings to the public to avoid alcohol industry-funded information sources should form an essential part of the global effort to tackle health misinformation.

**Supplementary Information:**

The online version contains supplementary material available at 10.1186/s12992-025-01125-4.

## Introduction

Access to health information is a crucial component of global population health promotion, with accurate, reliable, and up-to-date content contributing to collective and individual decision making that saves lives [1, 2]. Ensuring universal access to such information requires preventing undue commercial influence. Whilst commercial entities can contribute in positive ways to public health, health-harming industries such as alcohol, tobacco, and ultra-processed food and drink producers are known to try to reframe the health effects of their products in various ways, including by providing health information to the public [3, 4]. Such ‘education’ activities support wider efforts to frame not only the products, but also the industries themselves as socially responsible [4] and were first developed to avoid policies that would negatively impact on profits [5]. This shared ‘playbook’ of practices is one component of what are known as the commercial determinants of health, with profound effects on the extent to which the harms from such products, and the companies producing them, are perceived and addressed [3].

The alcohol industry provides health information to the public largely via corporate social responsibility organisations, which include industry-funded charities and nonprofit companies known as ‘social aspects and public relations organisations’ (SAPROs). Part of what distinguishes SAPROs from other industry companies and organisations is their efforts to present themselves as ‘independent’ charities (for example, [6]) and nonprofit companies (for example, [7]) despite being almost entirely funded by the alcohol industry. These efforts appear successful; there is evidence from Australia that the wider public believes these groups are government-funded [8]. As such, SAPROs are framed as ‘part of the solution’ to alcohol harms, whilst performing strategic functions for the industry as part of wider corporate political activities [9]. Drawing on behavioural economics and marketing literatures, Petticrew et al. [10] found that alcohol industry SAPROs utilise cognitive biases to steer consumers towards industry misinformation. This includes techniques of ‘dark nudges’, which encourage alcohol consumption (for example via social norming or priming of alcohol) and sludge, which makes health information difficult to access and behaviour change harder (for example positioning material in ways difficult to find and reducing the functionality of education materials) [10]. Previous studies have found that the information provided by these industry groups misrepresents the evidence on alcohol harms in relation to cancer [11], cardiovascular health [12], and pregnancy and infant health [13].

The teratogenic effects of alcohol are widely known and uncontroversial [14]. When consumed during pregnancy, alcohol can cross the placenta and cause severe abnormalities within the brain, heart, kidney, liver, gastrointestinal tract, and the endocrine systems [15]. The effects of prenatal alcohol exposure are greater than tobacco use and exposure to other hazards such as lead and radiation [14], and include a range of lifelong conditions known as foetal alcohol spectrum disorder (FASD) [16]. There is uncertainty surrounding the effects of low level alcohol consumption on pregnancy and infant health, where different results are found depending on the study design and types of outcome assessed [14]. Yet, no safe threshold of alcohol consumption during pregnancy has been identified [17] and consistent with many national guidelines, the UK guidance recommends that women do not drink during pregnancy and when trying to conceive [18]. Despite this common precautionary approach, a previous study found that alcohol industry-funded organisations are less likely than public health websites to state that no amount of alcohol is safe in pregnancy [13]. Like other industries such as tobacco [4], they are also more likely to emphasise uncertainties about the science on alcohol and to use ‘alternate causation’ (non-alcohol causes of alcohol harms) arguments in their website content [13].

A recent survey conducted in Australia found that pregnant women received the majority of information about alcohol use during pregnancy from written/electronic information and health providers, and to a lesser extent, family and friends [19]. The type of information was associated with alcohol use; women who consumed alcohol during pregnancy versus abstainers were more likely to obtain information on alcohol from written/electronic sources (the definition of which included industry). The most commonly recalled source of information was written/electronic information, therefore it is imperative the information they receive supports informed decision making about alcohol [19]. It is important to revisit and review the content, accuracy and framing of industry pregnancy and infant health information because it can change following public health-informed scrutiny [20]. The study by Lim et al. that found misinformation on industry websites (including SAPROs) [13] received extensive media coverage (for example, 21) and led to responses by three of the SAPROs studied in the form of rebuttal letters to the journal editor disputing the findings [22–24]. It is reasonable to assume that the other SAPROs studied were aware of the findings, considering that they provide information on alcohol (which requires being up-to-date on the latest evidence) and also considering wider surveillance and monitoring of scientific outputs by the alcohol and other health-harming industries [25, 26]. To our knowledge, there is no longitudinal research tracking how industry responds to independent analysis of their health-related content. The aim of the study was therefore to critically analyse pregnancy and infant health information on alcohol-industry funded organisation websites following the previous independent analysis by Lim et al. [13]. MP, MvS, and NM conceptualised the idea for and were co-authors on the previous study.

## Methods

### Study design

This study is a qualitative, critical analysis of alcohol industry information on alcohol, pregnancy and infant health. Critical social research acknowledges and centres the interrelationship between data, theory, pre-existing knowledge, and socio-political context [27]. Using this perspective, we analyse how industry-funded information on pregnancy and infant health connects to wider social and political structures that shape public health [27, 28]. We focus on SAPROs due to their prominent role in ‘education’ campaigns that present alcohol as a user, rather than a product, problem [29–31]. It is reasonable to expect people to access these websites for unbiased information, since they are portrayed as non-profit and ‘independent’ organisations that aim to reduce alcohol harms.

We studied the seven SAPROs originally included in the prior analysis by Lim et al. [13]:

1. The Association of Alcohol Responsibility and Education (Aware.org, referred to throughout as Aware) (South Africa; formerly ARA).
2. Éduc’alcool (Canada).
3. Foundation for Advancing Alcohol Responsibly (FAAR) (USA; formerly The Century Council).
4. Drinkaware IE (Ireland).
5. Drinkaware UK (United Kingdom).
6. DrinkWise (Australia).
7. International Alliance for Responsible Drinking (IARD) (global; formerly ICAP).

We note that the aim of the study was to analyse industry health messaging following independent analysis, rather than directly compare current findings using the same methods as Lim et al. [13].

### Data collection

Two authors (GM and CB) used a combined protocol to access pregnancy and infant health written information on the included websites. First, the menu tool on the homepage of each SAPRO website was used to search for relevant information. A combination of screenshots, saving pages as PDFs (where possible), web archiving, and written notes were used to document the user ‘journey’ through to pregnancy and infant health information. We accessed pages if the links included one or more of the following terms in the page title: pregnancy, fertility, breastfeeding, FASD/fetal alcohol spectrum disorder, prenatal alcohol exposure, miscarriage, and infant health. We also accessed pages on the effects of alcohol on the body. Secondly, we used the site search tool (where available) to search for the following terms: pregnancy, fertility, breastfeeding, FASD, prenatal alcohol exposure, fetal/foetal alcohol spectrum disorder, miscarriage, and infant health. Quotation marks were not used as this was not indicated as an instruction on any website. ‘Suggested terms’ were used when identified by the tools.

We piloted these methods by analysing information from the Drinkaware UK website. Based on this pilot, we used searches to identify one main page on pregnancy, and infant health on each site for in-depth analysis to avoid collecting volumes of irrelevant data or webpages on another topic where relevant content represented a small sub-section. We defined ‘pregnancy and infant health information’ as information on specific risks and/or advice about alcohol, pregnancy and infant health that appeared to be aimed at consumers rather than scientists, policymakers, and/or industry. This meant we excluded scientific reviews or summaries of guidance on the topic in different countries, for example. Where we could not identify a main page, we used relevant subcontent from a page which was primarily focussed on another topic, where available.

### Data analysis

Our thematic analysis comprised three steps. First, we repeated the analysis developed by Lim et al. [13] to examine the prevalence of accurate information and misinformation across the websites (see Table 2). We then conducted a second analysis, to compare the stated aims or purpose of the SAPRO with the amount of pregnancy and infant health information we could identify on the website (see Table 1) to examine the relationship between the two: that is, were the organisations’ informational practices consistent with its implied purpose? Thirdly, we used the ‘dark nudges and sludge’ misinformation framework developed by Petticrew et al. [10] to conduct an in-depth analysis of what we identified as the main pregnancy and infant health webpages on each website (or sub-section, if a main page could not be located) to examine those forms of misinformation that distort and/or undermine access to information. All data were independently coded by GM and CB using a combination of Microsoft Word, Excel, and highlight and comment functions in Adobe PDF reader and then second coded (checked by the other author). Any disagreements about the application of the framework were resolved by regular discussion by email or video call. The data were then combined into key themes by GM and reviewed by MP. The authors are experienced public health researchers with expertise in alcohol industry corporate political activity, and MP, MvS, and NM conceptualised the idea for and were co-authors on the previous study of industry information on pregnancy and infant health. This expertise and experience informed our interpretation and analysis. In line with previous studies of alcohol industry misinformation [11, 13], ethical approval was not required for our secondary analysis of publicly available data and documents. We used the SRQR reporting guidelines in the reporting of this study [32].

**Table 1:** Stated aims/purpose of seven SAPRO websites and extent of identified pregnancy and infant health information to the consumers

	Drinkaware UK	Éduc’alcool	DrinkWise	Drinkaware IE	Aware	FAAR	IARD
Example description of content available/purpose of the organisation identified whilst searching for pregnancy and infant health information	*‘Understanding the facts about how alcohol affects your mind and body can* *be the first step to increase your alcohol awareness and reduce your drinking…find all the facts you need here’ (SF11)*	*‘A good understanding of the facts will help you make better decisions when it comes to your* *own alcohol consumption…we’ve* *got it covered’ (SF10)*	*‘Useful topics: facts about drinking*,* alcohol and your health*,* support services’ (SF12*,* screenshot 1)*	*‘Drinkaware is the national charity working to prevent and reduce alcohol misuse in Ireland’ (SF7)* *‘We’re here to help you make informed choices about alcohol to protect health and wellbeing’ (SF7)*	*‘Aware.org educates* *identified audiences about the risks associated with* *irresponsible consumption of alcohol with a view to* *changing attitudes and behaviours to reduce alcohol related* *harm’ (SF13)*	*‘Empowering adults to make a lifetime of responsible alcohol choices as part of a balanced lifestyle’ (SF8)*	*‘We are the International Alliance for Responsible Drinking (IARD)*,* a not-for-profit organization dedicated to reducing harmful drinking and promoting understanding of responsible drinking (SF9)*
Any pregnancy and infant health information for consumers found on site? *	Yes	Yes	Yes	Yes	Yes	None identified	None identified
At least one webpage where the main subject is pregnancy and infant health information for consumers?	Yes (SF1)	Yes (SF5)	Yes (SF4)	None identified	Yes (SF3)	None identified	None identified
If no dedicated page identified, pregnancy and infant health information for consumers found elsewhere on site?	n/a	n/a	n/a	Yes (SF2)	n/a	None identified	None identified
**Information identified on main pregnancy and infant health page (or elsewhere on site if no main page available) on alcohol and**:							
Fertility	Yes (SF1)	Yes (SF5)	Yes (SF4)	None identified	Yes (SF3)	None identified	None identified
Miscarriage	Yes (SF1)	Yes (SF5)	Yes (SF4)	None identified	None identified	None identified	None identified
Pregnancy	Yes (SF1)	Yes (SF5)	Yes (SF4)	Yes (SF2)	Yes (SF3)	None identified	None identified
Breastfeeding	Yes (link to information elsewhere on site) (SF1)	Yes (SF5)	Yes (SF4)	None identified	None identified	None identified	None identified
Foetal alcohol spectrum disorder	Yes (SF1)	Yes (SF5)	Yes (SF4)	Yes (SF2)	Yes (SF3)	None identified	None identified

*Defined as information on specific risks and/or advice about alcohol, pregnancy, and infant health that appears to be aimed at consumers (rather than scientists, policymakers, and/or industry, for example)

**For details of webpages referenced, please see supplementary material 1

**Table 2:** Search results on seven SAPRO websites for key pregnancy and infant health terms

Searches [in order of most number of relevant results across sites]	Drinkaware UK	DrinkWise	Drinkaware IE	Éduc’alcool	FAAR	IARD	AWARE
Search function available?	Yes	Yes	Yes	Yes [but not on homepage]	Yes	Yes	No
*Search 1: ‘Pregnancy’*							
Total number of results	16 (SF15)	12 (SF16)	4 (SF22)	18 (SF23)	4 (SF24)	14 (SF31)	n/a
Result 1 links to directly relevant information for consumers? *	No: ‘*Alcohol and pregnancy information*’ [rebuttal to Guardian coverage of Lim et al]’ (SF15)	Yes: *‘Doctors and DrinkWise remind mums-to-be to say no to alcohol* *as pandemic pregnancies soar*’ (SF16) [subcontent only]	Yes: *‘how does alcohol affect me’* (SF22)	No: *‘Quebecers and alcohol’* (SF23)	No: *‘what does it mean to drink responsibly’* (SF24) [link sends user to homepage]	No: *‘drinking guidelines for pregnancy and breastfeeding’* (SF31) [list of different guidelines worldwide]	n/a
Result 2 links to directly relevant information for consumers?	Yes: ‘*Alcohol and pregnancy*’ (SF15)	Yes: ‘*DrinkWise videos for education programs’* (SF16) [subcontent only]	Yes: *‘How can we help you?*’ (SF22) [although further clicks required to link to relevant content]	Yes: *‘Low risk drinking’* (SF23) [subcontent only]	No: *‘Meet this year’s #TalkEarly Parenting Blogger Team*’ (SF24)	No: *‘Letter to the editor: IARD responds to Lim et al.’s (2019) analysis of framing and completeness of information disseminated by alcohol industry-funded organizations’* (SF31)	n/a
Result 3 links to directly relevant information for consumers?	No: *‘letter to the editor: alcohol and pregnancy’* [rebuttal to Lim et al] (SF15)	Yes: *‘Choose to DrinkWise’* (SF16) [subcontent only]	No: *‘example’* (SF22)	No: *‘pregnancy and drinking’* (SF23) [Éduc’alcool poster with indecipherable wording. See also SF12, screenshot 6)	No: *‘a little thinking about drinking’* (SF24)	No: *‘fetal alcohol spectrum disorders’* (SF31) [scientific review]	n/a
Results in order of relevance?	No	Yes	Yes	No	No	No	n/a
Top 3 results include main relevant advice page on the topic, where available?	Yes	No	Yes	No	No main advice page identified	No main advice page identified	n/a
Total number of directly relevant results out of three	1	3	2	1	0	0	n/a
*Search 2: ‘fertility’*							
Total number of results	14 (SF15)	4 (SF18)	0 (SF22)	0 (SF23)	2 (SF25)	1 (SF33)	n/a
Result 1 links to directly relevant information for consumers?	Yes: *‘is alcohol harming your fertility?’* (SF15)	Yes: *‘pregnant*,* planning a pregnancy*,* or breastfeeding?*’ (SF18)	n/a	n/a	No: *‘5 ways to exercise your freedom this memorial day’* (SF25)	No: *‘Letter to the editor: IARD responds to Lim et al.’s (2019) analysis of framing and completeness of information disseminated by alcohol industry-funded organizations’* (SF33)	n/a
Result 2 links to directly relevant information for consumers?	Yes: *‘alcohol*,* fertility and pregnancy’* (SF15)	Yes: *‘the effects of alcohol on your body’* (SF18)[subcontent only]	n/a	n/a	No: *‘5 steps to exercising your freedom this Memorial Day’* (SF25)	n/a	n/a
Result 3 links to directly relevant information for consumers?	No: *‘how many units and calories are in gin?’* (SF15)	Yes: *‘alcohol on the flip side: know the risks’* (SF18) [subcontent only]	n/a	n/a	n/a	n/a	n/a
Results in order of relevance?	Yes	Yes	n/a	n/a	n/a	n/a	n/a
Top 3 results include main relevant advice page on the topic?	Yes	Yes	n/a	n/a	No main advice page identified	No main advice page identified	n/a
Total number of directly relevant results out of three	2	3	0	0	0	0	n/a
*Search 3: ‘breastfeeding’*							
Total number of results	Three (SF15)	11 (SF19)	5 (SF22)	1 (SF23)	1 (SF26)	4 (SF34)	n/a
Result 1 links to directly relevant information for consumers?	Yes: *‘alcohol and breastfeeding: how does it affect your baby?*’ (SF15))	Yes: *‘Doctors and DrinkWise remind mums-to-be to say no to alcohol* *as pandemic pregnancies soar’* [subcontent only] (SF19)	No: *‘guest blog Dr David Comerford - sometimes the simple prompts are the best’* (SF22)	Yes: *‘pregnancy and drinking: your questions answered’* (SF23)	No: *‘NBC’s ‘The Slap’ parents needed a plan’* (SF26)	No: *‘Drinking Guidelines for Pregnancy and Breastfeeding’* (SF34)[list of different guidelines worldwide]	n/a
Result 2 links to directly relevant information for consumers?	No: *‘what to do if you’re trying to become pregnant’* (SF15)	Yes: *‘DrinkWise videos for education programs’* (SF19) [subcontent only]	No: *‘promoting wellbeing through behaviour change - winter 2021 research briefing’* (SF22)	n/a	n/a	No: *‘Drinking Guidelines: General Population’* (SF34)	n/a
Result 3 links to directly relevant information for consumers?	No: *‘breast cancer symptoms’* (SF15)	Yes: *‘choose to DrinkWise’* (SF19) [subcontent only]	No: *‘alcohol and cancer’* (SF22)	n/a	n/a	No: *‘Drinking Guidelines: General Population’* (SF34)	n/a
Results in order of relevance?	Yes	Yes	n/a	Yes	n/a	Yes	n/a
Top 3 results include main relevant advice page on the topic?	Yes	No	No	Yes	No main advice page identified	No main advice page identified	n/a
Total number of directly relevant results out of three	1	3	0	1	0	0	n/a
*Search 4: ‘FASD’*							
Total number of results	3 (SF15)	12 (SF20)	1 (SF22)	0 (SF23)	65 (SF27)	2 (SF35)	n/a
Result 1 links to directly relevant information for consumers?	Yes: *‘Foetal alcohol spectrum disorder (FASD)’ (*SF15, p7)	Yes *‘Doctors and DrinkWise remind mums-to-be to say no to alcohol* *as pandemic pregnancies soar’* (SF20) [subcontent only]	Yes: *‘how does alcohol affect me’* (SF22) [subcontent only]	n/a	No: *‘BAC calculator: the virtual bar’* (SF27)	No: *‘Fetal Alcohol Spectrum Disorders’* (SF35) [scientific review]	n/a
Result 2 links to directly relevant information for consumers?	Yes: *‘is alcohol harming your fertility’* (SF15) [subcontent only]	Yes: *‘indigenous education resources and partnerships’* (SF20) [subcontent only]	n/a	n/a	No: *‘prevent underage drinking’* (SF27)	No: *‘Letter to the editor: IARD responds to Lim et al.’s (2019) analysis of framing and* *completeness of information disseminated by alcohol industry-funded* *organizations’* (SF35)	n/a
Result 3 links to directly relevant information for consumers?	Yes: *‘alcohol and pregnancy’* (SF15)	Yes: *‘DrinkWise for education programs’ (SF20)* [subcontent only]	n/a	n/a	No: *‘discussing alcohol with your college student’* (SF27)	No result	n/a
Results in order of relevance?	No	No	Yes	n/a	None relevant	None relevant	n/a
Top 3 results include main relevant advice page on the topic?	Yes	No	Yes	No	No main advice page identified	No main advice page identified	n/a
Total number of directly relevant results out of three	3	3	1	0	0	0	n/a
*Search 5: ‘fetal/foetal alcohol spectrum disorder’ (same results for both spellings unless otherwise stated)*							
Total number of results	465 (SF15)	12 (same results as for ‘FASD’; no results for ‘foetal’ [SF12, screenshot 4])	179 (SF22)	144 (SF23)	1 (SF28) [no result for ‘foetal’ spelling; SF12, screenshot 7]	113 (SF32)	n/a
Result 1 links to directly relevant information for consumers?	Yes: *‘Foetal alcohol spectrum disorder’ (FASD)* (SF15)	Yes *‘Doctors and DrinkWise remind mums-to-be to say no to alcohol* *as pandemic pregnancies soar’* (SF20) [subcontent only]	No: *‘alcohol consumption in Ireland’* (SF22)	No: *‘drinking during adolescence: the importance of circumstance’* (SF23)	No: *‘curbing high-risk impaired drivers: DWI courts are leading by example’* (SF28)	No: *‘Fetal Alcohol Spectrum Disorders’* (SF32) [scientific review]	n/a
Result 2 links to directly relevant information for consumers?	No: *‘Guest blog: an a-z guide to quitting alcohol’* (SF15)	Yes: *‘indigenous education resources and partnerships’* (SF20) [subcontent only]	Yes: *‘how does alcohol affect me?’* (SF22) [subcontent only]	No: *‘Production procedure for Alcohol and Health reports’* (SF23)	n/a	No: *‘Letter to the editor: IARD responds to Lim et al.’s (2019) analysis of framing and* *completeness of information disseminated by alcohol industry-funded* *organizations’* (SF32)	n/a
Result 3 links to directly relevant information for consumers?	Yes: *‘is alcohol harming your fertility’* (SF15) [subcontent only]	Yes: *‘DrinkWise for education programs’* (SF20) [subcontent only]	No: *‘alcohol and mental health’* (SF22)	No: *‘What role does genetics play in the way our bodies’* (SF23)	n/a	No: *‘Drinking and obesity’* (SF32)	n/a
Results in order of relevance?	No	No	No	None relevant	None relevant	Yes	n/a
Top 3 results include main relevant advice page on the topic?	Yes	No	Yes	No	No main advice page identified	No main advice page identified	n/a
Total number of directly relevant results out of three	2	3	1	0	0	0	n/a
*Search 6: ‘prenatal alcohol exposure’*							
Total number of results	465 (SF15)	3 (SF21)	177 (SF22)	1 (SF23)	1 (SF29)	113 (SF36)	n/a
Result 1 links to directly relevant information for consumers?	No: *‘alcohol and breastfeeding: how does it affect your baby?’* (SF15)	Yes: ‘*Doctors and DrinkWise remind mums-to-be to say no to alcohol* *as pandemic pregnancies soar’* (SF21)	No: *‘alcohol consumption in Ireland’* (SF22)	No: *‘alcohol and mental health’* (SF23)	No: *‘New Data on Pandemic Drinking Among Parents Underscores Need for Responsible Approaches’* (SF29)	No: *‘fetal alcohol spectrum disorders’* (SF36) [scientific review]	n/a
Result 2 links to directly relevant information for consumers?	Yes: *‘Foetal alcohol spectrum disorder* (FASD)’ (SF15)	Yes: *‘pregnant*,* planning a pregnancy*,* or breastfeeding?’* (SF21)	Yes: *‘how does alcohol affect me?’* (SF22) [subcontent only]	n/a	n/a	No: *‘Standards for online alcohol marketing channels’* (SF36)	n/a
Result 3 links to directly relevant information for consumers?	No: *‘Response to Lancet report on global alcohol forecasts’* (SF15)	Yes: *‘the effects of alcohol on your body’* (SF21)[subcontent only]	No: *‘alcohol and mental health’* (SF22)	n/a	n/a	No: *‘How to add safeguards to social media marketing’* (SF36)	n/a
Results in order of relevance?	No	No	No	None relevant	None relevant	Yes	n/a
Top 3 results include main relevant advice page on the topic?	Yes	Yes	Yes	No	No main advice page identified	No main advice page identified	n/a
Total number of directly relevant results out of three	1	3	1	0	0	0	n/a
*Search 7: infant health*							
Total number of results	352 (SF15)	0 (SF12, screenshot 2)	176 (SF22)	73 (SF23)	*1 (SF30)*	71 (SF37)	n/a
Result 1 links to directly relevant information for consumers?	No: *‘stepping outside for our mental health’* (SF15)	n/a	No: *‘alcohol consumption in Ireland’* (SF22)	No: *‘Production procedure for Alcohol and Health reports*’ (SF23)	No: *‘Can a Minecraft playdate be good for kids?*’ (SF30)	No: ‘*Alcohol and Pleasure: A Health Perspective (1999*)’ (SF37)	n/a
Result 2 links to directly relevant information for consumers?	No: *‘women’s health strategy call for evidence’* (SF15)	n/a	No: *‘alcohol and mental health’* (SF22)	No: *‘What role does genetics play in the way our bodies’* (SF23)	n/a	No: *‘Health Warning Requirements’* (SF37)	n/a
Result 3 links to directly relevant information for consumers?	No: *‘mental health: effects of alcohol on the brain’* (SF15)	n/a	No: *‘alcohol and heart health’* (SF22)	No: *It’s never too soon to talk to kids about drinking’* (SF23)	n/a	No: *‘Health Warning Requirements’* (SF37)	n/a
Results in order of relevance?	None relevant	n/a	None relevant	None relevant	None relevant	None relevant	n/a
Top 3 results include main relevant advice page on the topic?	No	No	No	No	No main advice page identified	No main advice page identified	n/a
Total number of directly relevant results out of three	0	0	0	0	0	0	n/a
*Search 8: ‘miscarriage’*							
Total number of results	2 (SF15)	3 [same results as ‘prenatal exposure’; SF12, screenshot 3]	0 (SF22)	1 (SF23)	0 (SF12, screenshot 8)	0 (SF12, screenshot 9)	n/a
Result 1 links to directly relevant information for consumers?	Yes: *‘alcohol and pregnancy: miscarriage risk’* (SF15)	No: ‘*Doctors and DrinkWise remind mums-to-be to say no to alcohol* *as pandemic pregnancies soar’* (SF12, screenshot 3)	n/a	Yes: *‘pregnancy and drinking: your questions answered’* (SF23)	n/a	n/a	n/a
Result 2 links to directly relevant information for consumers?	Yes: *‘Foetal alcohol spectrum disorder’* (FASD)’ (SF15) [subcontent only]	Yes: *‘pregnant*,* planning a pregnancy*,* or breastfeeding?’* (SF12, screenshot 3)	n/a	n/a	n/a	n/a	n/a
Result 3 links to directly relevant information for consumers?	n/a	Yes: *‘the effects of alcohol on your body’* (SF12, screenshot 3] [subcontent only]	n/a	n/a	n/a	n/a	n/a
Results in order of relevance?	Yes	No	n/a	Yes	n/a	n/a	n/a
Top 3 results include main relevant advice page on the topic?	Yes	Yes	n/a	Yes	n/a	n/a	n/a
Total number of directly relevant results out of three	2	2	0	1	0	0	n/a

*Directly relevant here means provides information on risks and/or advice on the specific topic searched for. For consumers means advice and/or information that appears to be aimed at consumers (rather than, for example, policy makers, industry, scientists)

**For details of webpages referenced, please see supplementary material 1

## Results

Omission of information, functionality problems, and the positioning and sequencing of information in ways that framed or obfuscated its meaning [10] were the most common forms of misinformation we identified across the seven websites. These types of misinformation were often mixed with (limited) relevant information and were most often found in combination. Below, we outline key omissions, including how the stated aims/purpose of the seven SAPROs did not align with the pregnancy and infant health information available on their websites, creating a ‘meta’ form of stimulus incompatibility [10]. We then describe the user experience of the search tool functions (where available), and the patterns of positioning and sequencing misinformation across the sites.

### Omission of, or limited pregnancy and infant health information

Omission of information on pregnancy and infant health was common across all seven sites. When searching for information, we identified statements across all sites describing either general aims of the organisation or indications of the types of information available (see Table 1).

In all cases, these statements gave the impression that the sites provided general information and advice about alcohol and health. It is therefore reasonable to assume this would include information about alcohol, pregnancy, and infant health for the consumer. As seen in Table 1, however, we could only find substantial pregnancy and infant health information for the consumer on four of the seven SAPRO websites studied (Drinkaware UK, DrinkWise, Éduc’alcool and Aware). On one site, we could only find one short paragraph on the topic (Drinkaware IE). We could not find any pregnancy and infant health information directed to the consumer on the remaining sites (FAAR and IARD). On the five sites where we found pregnancy and infant health information, all explicitly mentioned pregnancy and fetal alcohol spectrum disorder. We could not find information on miscarriage or breastfeeding on the Aware site, nor information on fertility, miscarriage, or breastfeeding on the Drinkaware IE site (see Table 1). This created a ‘meta’ form of stimulus incompatibility, where the reader is prompted to expect (via the purported aims of the organisations) general information about health, but there was limited information on three of the sites, and mixed levels of information on specific topics.

### Functionality: website search tools

A search tool was available for six of the seven websites (see Table 2). We found no search terms that consistently identified relevant information across all the websites. *Pregnancy* was the term that produced at least one directly relevant result (out of the top three results) across most websites (four out of seven), although the top result was not always relevant. We found no relevant results for the search term *infant health* across any of the sites. Éduc’alcool ’s search function was not visible on the homepage (SF23, p1).

For all sites where a search tool was available, the tool did not seem to identify common terms (e.g. *fetal alcohol spectrum disorder*) and produced results for each word instead, which produced a large number of irrelevant results. For example, on the FAAR site the only result for this term was ‘curbing high-risk impaired drivers: DWI courts are leading by example’, and on the Drinkaware UK site, 465 results were produced, with only two of the top three directly relevant. This also meant that results were often not in order of most to least relevant. Even where the tool identified a suggested term, the very large number of irrelevant results indicated that the search was still retrieving results based on individual words (for example, Drinkaware UK, SF15, pp.9–10). In one case (FAAR), the tool seemed to search for individual letters. For example, searching *fertility* produced results that highlighted the word *fertilized* (SF25, p1) and a search for *FASD* produced results that highlighted the words *fast* and *faster* (SF26, p1). On the Drinkaware UK and IARD websites, when searching ‘pregnancy’, each SAPROs rebuttal to Lim et al. [13] was the first (Drinkaware UK) and second (IARD) result. That is, health information aimed at the consumer came after a response to public health critique in Drinkaware UK’s case, and as part of three irrelevant (not directed towards consumer) results in the case of IARD.

### Positioning and sequencing of information

We identified positioning and sequencing misinformation across all five sites with pregnancy and infant health information. The order of information matters for three reasons: (1) the reader’s response to information on alcohol harm is shaped by the first thing they read/see, to the extent that subsequent information is mostly wasted; (2) isolated instances do not necessarily constitute misinformation, whereas repeated patterns do; and (3) it affects how the reader engages with and processes the information [10]. We identified several pregnancy and infant health pages on the Drinkaware UK website, although the main page with actual advice (rather than links to other pages, or general tips about cutting down on drinking) was quite difficult to find. For example, when clicking the link ‘health effects of alcohol’, fertility and pregnancy was sixth out of nine listed effects (SF14). Above that was a link to ‘alcohol and the body’, where we could find no relevant information, and a section on ‘alcohol and gender’. The latter did not provide any information on that page, but provided links to two further separate pages (one for men, one for women) which did have relevant information (SF14).

Patterns of positioning and sequencing misinformation were common at the webpage, paragraph, and even sentence level, with the most important information often coming last:

Through the evidence-based Australian guidelines to reduce health risks from drinking alcohol, the National Health and Medical Research Council (NHMRC) provide Australians with evidence-based advice on the health effects of drinking alcohol. The guidelines advise that to prevent harm from alcohol to their unborn child, women who are pregnant or planning a pregnancy should not drink alcohol. For women who are breastfeeding, not drinking alcohol is safest for their baby.’

(DrinkWise, ‘*Pregnant*,* planning a pregnancy or breastfeeding?*’ Accessed 11th August 2022 [SF4, p2])

The main pregnancy and infant health page on the Éduc’alcool site was situated on the sixth page of ‘facts and consequences’ (SF23, p9). A page on their website titled ‘pregnancy and drinking’, which was a result in several searches, was simply a poster resource labelled ‘can I raise a glass to my baby’s health?’ (SF12, screenshot 6), with the text sufficiently small as to be difficult to read at a distance.

### Mixing misinformation with relevant information

Part of what makes misinformation difficult to identify and analyse is that it can be mixed with relevant information (defined here as information on pregnancy and infant health aligned with the best available current evidence). This may be a deliberate strategy. Re-analysis of the content of the seven sites previously studied by Lim et al. (see Table 3) identified that relevant information was mixed with misinformation across the websites.

**Table 3:** Content of pregnancy and infant health information for consumers provided on seven SAPRO websites

Information type (based on Lim et al. 2019)	Drinkaware UK (based on main pregnancy and infant health page)	DrinkWise (based on main pregnancy and infant health page)	Éduc’alcool (based on main pregnancy and infant health page)	Aware (based on main pregnancy and infant health page)	Drinkaware IE [based on subsection of pregnancy and infant health information]	FAAR [no pregnancy and infant health information Identified]	IARD [no pregnancy and infant health information identified]
Relevant* information indicators							
Statement that no amount of alcohol is safe during pregnancy (not just first 3 months) [all in same sentence/two sentences]	Mixed [states not drinking is safest approach but over several sentences; SF1, p.1]	Not identified	Not identified	Yes (SF3)	Yes (SF2)	Not identified	Not identified
Units of reference (e.g. no. of units or drinks) provided regarding drinking in pregnancy	Yes (SF1)	Not identified	Yes (SF5)	Not identified	Not identified	Not identified	Not identified
Statement about risk of drinking during early stages of pregnancy (especially first trimester)	Yes (SF1)	Not identified	Yes (SF5)	Yes (SF3)	Not identified	Not identified	Not identified
Includes biological explanation of how alcohol affects the foetus during pregnancy	Yes (SF1)	Yes (SF4)	Yes (SF5)	Yes (SF3)	Yes (SF2)	Not identified	Not identified
Direct imperative used (e.g. ‘don’t drink’ or ‘stop’)	Yes (SF1)	Yes (SF4)	Not identified	Not identified	Not identified	Not identified	Not identified
*Misinformation indicators*							
Emphasis on individual choice and responsibility	Yes (SF1)	Mixed	Yes (SF5)	Mixed	Mixed	Not identified	Not identified
Use of language emphasising uncertainty (excluding statement that there is no evidence of safe amount during pregnancy)	Not identified	Not identified	Yes (SF5)	Not identified	Not identified	Not identified	Not identified
Use of alternative causation arguments to propose alternative causes of alcohol harms in pregnancy.	Yes (SF1)	Not identified	Yes (SF5)	Yes (SF3)	Not identified	Not identified	Not identified
Includes mixing modifiable with non-modifiable factors (e.g. drinking and ‘being small’)	Not identified	Not identified	Not identified	Not identified	Not identified	Not identified	Not identified
Presentation of evidence as ‘opinion’ or ‘belief’	Not identified	Not identified	Yes (SF5)	Not identified	Not identified	Not identified	Not identified
Presentation of ‘light’ or ‘moderate’ drinking and abstaining as equivalents	Not identified	Not identified	Yes (SF5)	Not identified	Not identified	Not identified	Not identified

*Relevant information is defined here as information on pregnancy and infant health aligned with the best available current evidence

**For details of webpages referenced, please see supplementary material 1

However, relevant information was limited; of the five relevant information indicators listed in Table 3, only one was found on all five sites (a biological explanation of how alcohol affects the foetus during pregnancy on their main pregnancy and infant health page):

Alcohol crosses from the mother’s blood stream into the baby’s blood stream and can affect the baby’s development. If you are pregnant and drink then so does your baby and that can cause harm.

(Drinkwise, ‘*‘Pregnant*,* planning a pregnancy or breastfeeding?’* Accessed 11th August 2022 [SF4, p2])

This kind of information was most often mixed in with the types of misinformation indicators outlined. For example, on the Drinkaware UK website, relevant information was sequenced in a way where the advice not to drink was positioned towards the end of the paragraph, and there was no direct imperative (e.g. ‘you should not drink alcohol’):

Drinking alcohol at any stage during pregnancy can cause harm to your baby – and the more you drink, the greater the risk. That’s why the UK Chief Medical Officers’ low risk drinking guidelines advise that if you’re pregnant or think you could become pregnant, the safest approach is not to drink alcohol at all, to keep risks to your baby to a minimum. Not drinking alcohol is the safest approach.

(Drinkaware UK, *‘Alcohol and pregnancy*’, accessed 28th July 2022 [SF1, p2])

Although ‘your baby’ was referenced, the last sentence included no direct instruction to the reader personally. Subtle differences in presentation can have a significant impact on how people perceive a problem and whether they think the information is relevant to them [10].

### Combinations of misinformation strategies

Although we have outlined the key types of misinformation separately above, they were commonly used in combination. Below, we provide three illustrative examples:

Q: I enjoy a glass of wine with a good meal. Do I have to change my habits during my pregnancy?

R: To date, researchers have not been able to determine the exact amount of alcohol that is completely safe for the development of the foetus, even though there is no evidence that the occasional drink has any harmful effect. We do know, however, that the risk of miscarriage, birth defects, growth retardation and mental disorders increases the more drinks the mother has on each occasion, and the more frequently she drinks. The scientific community believes that abstaining from drinking is the safest choice. In any case, you can always discuss your drinking with your doctor and get help if you need it.

(Éduc’alcool, ‘*Pregnancy and drinking: your questions answered*’, accessed 16th August 2022 [SF5, pp. 1–2])

Misinformation strategies used here include priming drinking (‘enjoy a glass of wine with a good meal’), positioning and sequencing (the advice to abstain is the second to last sentence), framing (‘in any case’ dilutes the risk and overall message provided), presenting scientific evidence as ‘belief’, encouraging uncertainty (‘researchers have not been able to determine the exact amount...there is no evidence...’) and omission (the comment that the risk of miscarriage and other harms increase the more a mother drinks alcohol is misleading, because it is not stated that any amount increases the risk).

The second example is a ‘tie the knot’ campaign by Aware (SF3, p1), which is ostensibly about FASD. The campaign is summarised in a large box with text and images, including of several stages of a knot being tied in what appears to be a piece of rope. The most prominent text includes:

Their tomorrow starts when you don’t drink.

How to tie the FASD knot: (1) Make a loop. (2) Right over left. (3) Left over right. (4) Finished knot [with images of knots].

(Aware, ‘*Fetal alcohol spectrum disorder*’, accessed 16th August 2022 [SF3, p.1]).

The only images are ‘knots’, and alcohol is not named in the most prominent text. This would appear to be a relatively complex way of saying that prenatal alcohol exposure can cause a range of lifelong conditions, known as FASD. A section titled ‘symbolism’ is required to explain what the images represent, implying that the images alone are insufficient for the reader to understand what the knot means. There is an explanation for each image of progressively tying a knot, with ‘the circle’ (described as ‘the loop’ elsewhere on the page) described as ‘the baby’s head, the human brain and the world’; ‘the cord’ described as ‘the umbilical cord and our ties to the community’, the ‘reef knot’ described as ‘the more you pull, the tighter the bond’, and ‘the frayed ends’ described as ‘the irreparably damaged nervous system’. The connections between each symbol and what they represent are not described, and we found it extremely difficult to connect these to any advice or specific awareness raising about FASD. We argue this imagery is a form of stimulus incompatibility – the campaign is purported to be about raising awareness of FASD, but the imagery is simply of a piece of what looks like rope tied in various stages of a knot.

The largest, beginning paragraph of detailed text highlights a partnership between Aware and the South African government, and is placed above information about FASD, which is a form of positioning and sequencing misinformation (the reader’s response to information on alcohol harm is shaped by the first thing they read/see, to the extent that subsequent information is mostly wasted [10]). There are also several omissions – the hashtag #awareoftomorrow contains no reference to alcohol or FASD, and there are no images of alcohol, pregnancy, or children to help the reader quickly understand the content of the page.

The third example is from the Drinkaware IE website:

Drinking alcohol during pregnancy can cause foetal alcohol spectrum disorders (FASD). The most serious FASD is foetal alcohol syndrome. Alcohol can damage the developing brain and body by passing from the mother’s blood into the baby’s blood through the placenta. The HSE [Health Service Executive] advises that there is no safe amount and no safe time for alcohol during pregnancy.

(Drinkaware IE, *‘How does alcohol affect me?’*, accessed 28th July 2022 [SF2, p.1])

This is the entirety of the information we could find on alcohol, pregnancy and infant health across the Drinkaware IE website, despite the aim of the organisation described as ‘we’re here to help you make informed choices about alcohol to protect health and wellbeing’ (SF7, p.3). This is a combination of omission and stimulus incompatibility; the reader is told that the aim of the site is to help make informed choices, but there is very little information on alcohol, pregnancy, and infant health. The advice that there is ‘no safe time and no safe amount’ is at the end of the paragraph, with no direct imperative, which is a form of positioning and sequencing misinformation This paragraph did appear several times in search results (see Table 1), and does mean the organisation can claim to provide information on the topic, even if this totals three sentences.

## Discussion

Omission of information, functionality problems, and the positioning and sequencing of information in ways that framed or obfuscated its meaning were the most common forms of ‘dark nudges and sludge’ misinformation [10] identified across the seven industry-funded websites. This was often mixed with (limited) relevant information, and different types of misinformation were usually used in combination. We found substantial pregnancy and infant health information directed towards the consumer on only four of the seven websites (Aware, Éduc’alcool, Drinkaware UK, and DrinkWise). On one further website, we could only find one short paragraph on the topic (Drinkaware IE). We could not find any pregnancy and infant health information directed to the consumer on the remaining sites (FAAR and IARD). We could not find information on miscarriage or breastfeeding on the Aware site, nor information on fertility, miscarriage, or breastfeeding on the Drinkaware IE site.

Our findings show that misinformation about alcohol, pregnancy, and infant health continues on SAPRO websites. The types of misinformation identified here are often subtle, and it is the pattern both across and within the websites that demonstrates a lack of effective measures taken by the industry or its funded organisations to address the issue. This is despite a previous, independent study highlighting the problem in a peer-reviewed, academic journal [13], which received media coverage [21] and negative responses from the industry [22–24]. Given that these SAPROs are funded by a trillion-dollar industry [33] that claims to be acting to address the harms associated with alcohol use, the ongoing dissemination of misinformation is inexcusable and contradicts the stated aims and purpose of the organisations they fund. Similarly, the ‘sludge’ we identified [10] via the poor functionality across the websites and how difficult (or impossible) we found it to identify pregnancy and infant health information cannot reasonably be explained by a lack of resources. Most consumers will not use our methods [10], therefore it is likely that they receive even less information on detailed aspects of pregnancy and infant health than we identified.

There were some differences in our findings compared to previous analysis. Lim et al. [13] found that industry websites were less likely than public health websites to provide information on FASD, whereas we found at least some information on five of the seven websites, although in the case of DrinkWise and Drinkaware IE, this information was minimal. We also did not find information on breastfeeding, fertility, or miscarriage on every site. Our methodology differed in places to Lim et al., which could account for these findings. A recent study exploring alcohol, pregnancy, and infant health information on Twitter (now X) found a similar focus on FASD and less on other topics such as miscarriage across industry and non-industry websites [34], suggesting a focus on FASD may not be confined to industry sources.

A key component of misinformation is that it can be mixed in with (limited) relevant information. Where this occurs, it is a function of misinformation, rather than an exception to it, and it may echo the shift Mann [35] describes of fossil fuel companies moving from ‘hard’ to ‘soft’ denialism because outright denial of climate change is no longer tenable. Health-harming industries are known to share the same ‘playbook’ of strategies [4]; outright denials that alcohol is not harmful are also not credible [36]. Instead, a growing body of evidence suggests a whole population approach to prenatal alcohol exposure is required that also addresses alcohol consumption by non-pregnant women and men [14] and provides the long overdue recognition of pregnancy among transgender non-binary, and gender-expansive people [37]. Creating health environments that support healthy pregnancies is key to any prevention agenda. Further, continuing to allow and enable the dissemination of industry-funded information as a source of advice about alcohol, pregnancy, and infant health risks undermining the promotion of women’s health and exacerbating gender or social inequities in access to high quality health promotion.

The documentation of not only the misinformation on the sites, but the process by which a user consumes that content is a strength of the study, because it allowed us to systematically identify omissions. Public health actors have argued that monitoring and exposing corporate activities should comprise part of the global ‘public health playbook’ [38] to help counter industry activity that negatively impacts health [39, 40]. Based on our findings, this should include more longitudinal analysis that identifies how industry adapts and responds to independent analysis. Limitations include only studying seven SAPROs; there are many more industry organisations globally providing pregnancy and infant health information, and study of those would help identify any differences between various types of industry groups. Content on the sites is likely to have changed since we collected the data, although our findings indicate that the passage of time does not lead to the removal of misinformation across the sites. Our study is limited to written information on the sites, as we excluded video content for reasons of feasibility/capacity. We did not compare industry-funded information to public health actor information, as the latter had previously been found not to promote misinformation [13]. This could mean we missed an opportunity to identify any omissions on particular topics by non-industry sources.

While health misinformation is not new [41], what is new is the sheer size and scale of transnational companies [33] and the power that gives them to allocate resources to promote commercial rather than public health interests. Alcohol companies fund SAPROs to support a framing of the industry as an essential economic and social actor and force for good [4, 42]. Our findings demonstrate the opposite, and add to recent studies of other alcohol industry SAPRO activities that are in direct opposition to public health: the wider promotion of misinformation [11, 12, 43]; efforts to promote ineffective self-regulation measures rather than effective interventions to address alcohol harms [44]; public-private (SAPRO) partnerships that result in interventions that do not align with evidence-based recommendations [28, 45–47]; and alcohol industry youth education programmes, which were found to promote moderate alcohol consumption and industry interests in several countries regardless of whether they were sponsored by a SAPRO, alcohol company, or charity with extensive connections to the alcohol industry [48].

On the basis of this growing body of evidence, we add to existing calls for public health actors to both stop referring people to industry-funded sources for supposed health information and to avoid any health-information related partnerships with SAPROs, and for the public to be warned to avoid such sources due to their potential for harm [11–13, 43]. We also argue that more should be done to raise awareness of who funds SAPROs, as there is evidence from Australia that the wider public believes such organisations are government-funded [8]. The provision of high-quality health information – a key pillar of universal health coverage – should not be outsourced to health-harming industries like the alcohol industry whose interests are in direct conflict with global health and equity goals. Alcohol industry SAPROs should be regulated as the industry-funded and industry-friendly organisations they are, rather than as the ‘independent’ charities and not-for-profit companies they present themselves to be.

## Electronic supplementary material

Below is the link to the electronic supplementary material.

Supplementary Material 1

## Data Availability

The data analysed during the current study are not publicly available due to copyright restrictions. Reviewers had access to all data cited in the form of supplementary files.
