# Supplementary material for: Do alcohol industry-funded organisations act to correct misinformation? A qualitative study of pregnancy and infant health content following independent analysis
Source: Global Health. 2025 Nov 12;21:68. doi: 10.1186/s12992-025-01125-4 (PMC12613373; doi:10.1186/s12992-025-01125-4)
Supplement: Supplementary file 1 — Supplementary Material 1 [file 12992_2025_1125_MOESM1_ESM.docx]

| **Document number** | **Social aspects and public relations organisation (SAPRO)** | **Description** | **Date accessed** |
| --- | --- | --- | --- |
| 1 | Drinkaware UK | ‘alcohol and pregnancy’ webpage | 28^th^ July 2022 |
| 2 | Drinkaware IE | ‘How does alcohol affect me’ webpage | 28^th^ July 2022 |
| 3 | Aware | ‘Fetal alcohol spectrum disorder’ webpage | 16^th^ August 2022 |
| 4 | Drinkwise | ‘Pregnant, planning a pregnancy or  breastfeeding?’ webpage | 11^th^ August 2022 |
| 5 | Educ’alcool | ‘Pregnancy and drinking: your questions answered’ webpage | 16^th^ August 2022 |
| 6 | Drinkaware UK | Homepage | 28^th^ July 2022 |
| 7 | Drinkaware IE | Homepage | 28^th^ July 2022 |
| 8 | FAAR | Homepage | 24^th^ August 2022 |
| 9 | IARD | Homepage | 16^th^ August 2022 |
| 10 | Educ’alcool | ‘Facts and consequences’ webpage | 16^th^ August 2022 |
| 11 | Drinkaware UK | ‘Facts about alcohol’ | 26^th^ July 2022 |
| 12 | Several | Screenshot 1: Drinkwise homepage  Screenshot 2: Drinkwise search result ‘infant health’  Screenshot 3: Drinkwise ‘miscarriage’ search results  Screenshot 4: Drinkwise ‘foetal alcohol spectrum disorder’ search result  Screenshot 5: Aware homepage  Screenshot 6: Educ’alcool, ‘Pregnancy and Drinking’ webpage  Screenshot 7: FAAR search result ‘foetal alcohol spectrum disorder’  Screenshot 8: FAAR search results ‘miscarriage’  Screenshot 9: IARD search results ‘miscarriage’ | 15^th^ August 2022  16^th^ August 2022  16^th^ August 2022  16^th^ August 2022  16^th^ August 2022  16^th^ August 2022  24^th^ August 2022  24^th^ August 2022  19^th^ August 2022 |
| 13 | Aware | ‘We are Aware’ webpage | 16^th^ August 2022 |
| 14 | Drinkaware UK | ‘Health effects of alcohol’ webpage | 26^th^ July 2022 |
| 15 | Drinkaware UK | All search results (listed and screenshots where available) | 28^th^ July 2022 |
| 16 | Drinkwise | ‘Pregnancy’ search (full list of results on single webpage) | 15^th^ August 2022 |
| 17 | Drinkwise | ‘Pregnancy’ search result 1 | 15^th^ August 2022 |
| 18 | Drinkwise | ‘Fertility’ search (full list of results on single webpage) | 15^th^ August 2022 |
| 19 | Drinkwise | ‘Breastfeeding’ search (full list of results on single webpage) | 15^th^ August 2022 |
| 20 | Drinkwise | ‘FASD’ search (full list of results on single webpage) | 16^th^ August 2022 |
| 21 | Drinkwise | ‘Prenatal alcohol exposure’ search (full list of results on single webpage) | 16^th^ August 2022 |
| 22 | Drinkaware IE | All search results (listed and screenshots where available) | 29^th^ July 2022 and 11^th^ August 2022 |
| 23 | Educ’alcool | All search results (listed and screenshots where available) | 16^th^ August 2022 |
| 24 | FAAR | ‘Pregnancy’ search (full list of results on single webpage) | 24^th^ August 2022 |
| 25 | FAAR | ‘Fertility’ results (full list of results on single webpage) | 24^th^ August 2022 |
| 26 | FAAR | ‘Breastfeeding’ results (full list of results on single webpage) | 24^th^ August 2022 |
| 27 | FAAR | ‘FASD’ results (full list of results on single webpage) | 24^th^ August 2022 |
| 28 | FAAR | ‘Fetal alcohol spectrum disorder’ search (full list of results on single webpage) | 24^th^ August 2022 |
| 29 | FAAR | ‘Prenatal alcohol exposure’ search (full list of results on single webpage) | 24^th^ August 2022 |
| 30 | FAAR | ‘Infant health’ search (full list of results on single webpage) | 24^th^ August 2022 |
| 31 | IARD | ‘Pregnancy’ search (page 1 of results on single webpage) | 19^th^ August 2022 |
| 32 | IARD | ‘ Fetal alcohol spectrum disorder’ search (full list of results on single webpage) | 19^th^ August 2022 |
| 33 | IARD | ‘Fertility’ results (full list of results on single webpage) | 19^th^ August 2022 |
| 34 | IARD | ‘Breastfeeding’ results (full list of results on single webpage) | 19^th^ August 2022 |
| 35 | IARD | ‘FASD’ search (full list of results on single webpage) | 19^th^ August 2022 |
| 36 | IARD | ‘Prenatal alcohol exposure’ search (full list of results on single webpage) | 19^th^ August 2022 |
| 37 | IARD | ‘Infant health’ search (full list of results on single webpage) | 19^th^ August 2022 |
